# Modulation of Mucin (*MUC2, MUC5AC* and *MUC5B*) mRNA Expression and Protein Production and Secretion in Caco-2/HT29-MTX Co-Cultures Following Exposure to Individual and Combined Aflatoxin M1 and Ochratoxin A

**DOI:** 10.3390/toxins11020132

**Published:** 2019-02-23

**Authors:** Xin Huang, Yanan Gao, Songli Li, Chenqing Wu, Jiaqi Wang, Nan Zheng

**Affiliations:** 1 Key Laboratory of Quality & Safety Control for Milk and Dairy Products of Ministry of Agriculture and Rural Affairs, Institute of Animal Sciences, Chinese Academy of Agricultural Sciences, Beijing 100193, China; 15501852343@163.com (X.H.); gyn758521@126.com (Y.G.); Lisongli@caas.cn (S.L.); wuchenqing111@163.com (C.W.); wangjiaqi@caas.cn (J.W.); 2 Laboratory of Quality and Safety Risk Assessment for Dairy Products of Ministry of Agriculture and Rural Affairs, Institute of Animal Sciences, Chinese Academy of Agricultural Sciences, Beijing 100193, China; 3 Milk and Milk Products Inspection Center of Ministry of Agriculture and Rural Affairs, Institute of Animal Sciences, Chinese Academy of Agricultural Sciences, Beijing 100193, China; 4 State Key Laboratory of Animal Nutrition, Institute of Animal Science, Chinese Academy of Agricultural Sciences, Beijing 100193, China

**Keywords:** aflatoxin M1, ochratoxin A, Caco-2/HT29-MTX co-cultures, mucin, interactive effects

## Abstract

Aflatoxin M1 (AFM1) and ochratoxin A (OTA), which widely coexist in milk, may pose a serious threat to human health. Mucin is a major component of the intestinal mucus layer, which plays an important role in maintaining intestinal mucosal homeostasis. However, the effect of mycotoxins AFM1 and OTA on intestinal mucin production is still not clear. This study aimed to investigate individual and interactive effects of mycotoxins AFM1 and OTA on the intestinal barrier and the mRNA expression of intestinal mucin (*MUC2*, *MUC5AC* and *MUC5B*) and on protein production in Caco-2/HT29-MTX cultures after 48 h of exposure. Our results show that individual mycotoxins and their mixtures significantly reduced intestinal cell viability and transepithelial electrical resistance (TEER) values, as well as significantly altered intestinal mucin mRNA expression and protein abundance. Moreover, OTA showed toxicity similar to AFM1 in cell viability and TEER value at the same concentration. When the two mycotoxins acted in combination, the synergistic effects observed in the assessment of cell viability and protein abundance in all mono- and co-cultures. In general, this study provides evidence that AFM1 and OTA can damage the intestine, and it contributes to optimized maximum permissible limits of mycotoxins in milk.

## 1. Introduction

Mycotoxins are structurally diverse low-molecular-weight metabolites produced by the secondary metabolism of some filamentous fungi or molds [1]. They can contaminate a variety of animal feed, as well as foods for humans, including mostly cereals, milk, and other dairy products [2]. A report suggested that as much as 50% of commodities may be contaminated by mycotoxins in certain situations [3]. Furthermore, mycotoxins tend to persist during the processing of contaminated foods and are usually not eliminated during cooking and sterilization [4]; hence, food contamination by mycotoxins has been recognized as a public health threat [2]. Cow milk consumption is high because it is important in the diet of all age groups [5]. The coexistence of mycotoxins in milk and dairy products has recently attracted much attention, especially the coexistence of aflatoxins (AFs) and ochratoxin A (OTA) [6].

It has been reported that aflatoxin M1 (AFM1) and OTA are major risk factors in milk that may pose a threat to human health [7]. AFM1, a metabolite of AFB1, is the only mycotoxin with an established maximum residue limit (MRL) in milk worldwide. The established MRL of AFM1 is 0.05 μg/L in the European Union, while it is 0.5 μg/L in China and the United States [8]. AFM1 cannot only contribute to the causation of liver cancers, immune system disorders, and growth-related issues in children [9], but it also causes damage to the intestinal barrier such as injury to intestinal cells, destruction of intestinal tight junctions, and an increase in intestinal permeability [10]. OTA is a stable compound produced by *Aspergillus* and *Penicillium* [11]. It is not destroyed by common food preparation procedures, and temperatures above 250 °C for several minutes are required to reduce its concentration [12]. According to reports, OTA is hepatotoxic, nephrotoxic, immunotoxic and teratogenic in animals [1]. In addition, OTA is cytotoxic to the intestinal epithelium and the mucosa-associated lymphoid tissue, altering the intestinal barrier and increasing susceptibility to various associated diseases [13]. Considering that multi-exposure to mycotoxins is the most likely scenario and that co-occurrence of mycotoxins can affect their toxicological effects on humans and animals [5], we find it necessary to determine the combined cytotoxicity of AFM1 and OTA.

A dynamic, well-regulated intestinal barrier is crucial for protecting the body from dietary antigens and residing intestinal microbiota [11]. This barrier is mainly composed of intestinal epithelial cells, symbiotic microbial communities, and the intestinal mucus. The intestinal mucus forms a single, easily removable layer in the small intestine or forms a double layer in which the inner mucus layer is firmly attached to the epithelium in the colon [14]. Besides, a new concept was proposed that the inner mucus layer is attached to the fecal pellet in the distal colon of rodents, confining the microbiota to the faeces [15]. The mucus layer in the gastrointestinal tract acts as the first line of defense against threats such as mycotoxins and as an environment that is beneficial to endogenous symbiotic microbiota [16]. Evidence has shown that the presence or absence of mucin secreted by goblet cells in the gastrointestinal tract or the up- and down-regulation are related to gastrointestinal inflammation and related diseases and even cancer [17,18]. However, up to date, limited data reveal that mycotoxins cause alterations in intestinal mucin expression and secretion, although there is much evidence from in vivo and in vitro models indicating that mycotoxins can cause intestinal damage [19,20,21]. Thus, it is important to evaluate the impact of AFM1 and OTA and their interactions on potential toxicological targets in the intestine, including mucin synthesis and secretion.

One of the commonly used intestinal model, HT29-MTX cells are a homogeneous subpopulation of HT29 human colon carcinoma cells selected by adaptation to 10^−5^ M methotrexate, they produce mucins, in particular, MUC2, MUC5AC, and MUC5B; as such, they can be regarded as providing some similar function to goblet cells in mucin secreting [22]. Although mucin is mainly secreted by HT29-MTX cells, co-culture models combining HT29-MTX with Caco-2 cell lines at ratios that represent the small (90/10 for Caco-2/HT29-MTX) and large intestine (75/25 for Caco-2/HT29-MTX) were chosen in order to mimic closely the permeability features of the human intestinal barrier [23]. We then characterized the impact of individual and combined AFM1 and OTA on mucin (*MUC2*, *MUC5AC* and *MUC5B*) mRNA expression and protein production and secretion. As far as we know, this is the first time that intestinal cell co-culture models were used to assess the interaction between AFM1 and OTA. We proved that AFM1 and OTA not only significantly damaged the intestinal cell viability and increased intestinal permeability, but also changed the expression and secretion of mucin.

## 2. Results

### 2.1. Effects of AFM1 and OTA Individually Or in Combination on Caco-2/HT29-MTX Cell Viability (100/0, 90/10, 75/25, and 0/100)

**Figure 1:**
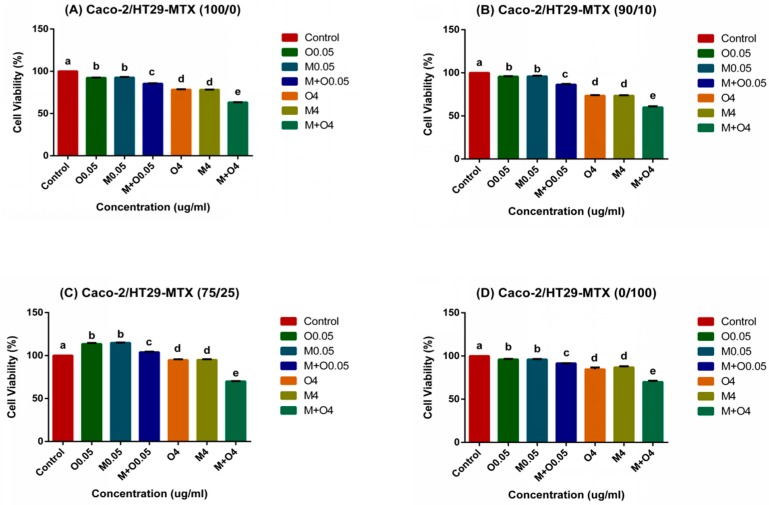
Effects of individual and mixtures of aflatoxin M1 (AFM1) and ochratoxin A (OTA) on cell viability of Caco-2/HT29-MTX (**A**) 100/0, (**B**) 90/10, (**C**) 75/25, and (**D**) 0/100 co-cultures. Differentiated Caco-2/HT29-MTX cells were exposed to AFM1 (0.05, 4 μg/mL), OTA (0.05, 4 μg/mL), or OTA+AFM1 (0.05, 4 μg/mL) for 48 h, then cell viability was determined by using the enhanced Cell Counting Kit-8 (CCK-8). Results are expressed as percentage of control and are means ±SEM (*n* = 3), respectively. Different letters (a–e) indicate significant differences in cell viability (*p* < 0.05). M0.05 represents AFM1 at 0.05 μg/mL, M4 represents AFM1 at 4 μg/mL, O0.05 represents OTA at 0.05 μg/mL, O4 represents OTA at 4 μg/mL, M+O0.05 represents AFM1+OTA at 0.05 μg/mL, M+O4 represents AFM1+OTA at 4 μg/ mL.

Incubation with individual AFM1 and OTA and their mixtures at 0.05 and 4 μg/mL for 48 h significantly reduced the cell viability (*p* < 0.01) to about 60%. Furthermore, the mixtures of AFM1 and OTA at 4 μg/mL caused the most obvious damage to Caco-2/HT29-MTX mono- and co-cultures when compared with the control group (Figure 1). In the 75/25 co-cultures, mycotoxins at lower concentrations (0.05 μg/mL) stimulated the increase in cell viability values (Figure 1C). At the same concentration, the toxic effect of AFM1 combined with OTA was significantly higher than that of either alone (*p* < 0.01) (Figure 1). Moreover, AFM1 and OTA alone had similar cytotoxicities and there was no significant difference (*p* > 0.05, Figure 1).

### 2.2. Effect of AFM1 and OTA in Combination on Caco-2/HT29-MTX Cell Layer Structures (100/0, 90/10, 75/25, and 0/100)

**Figure 2:**
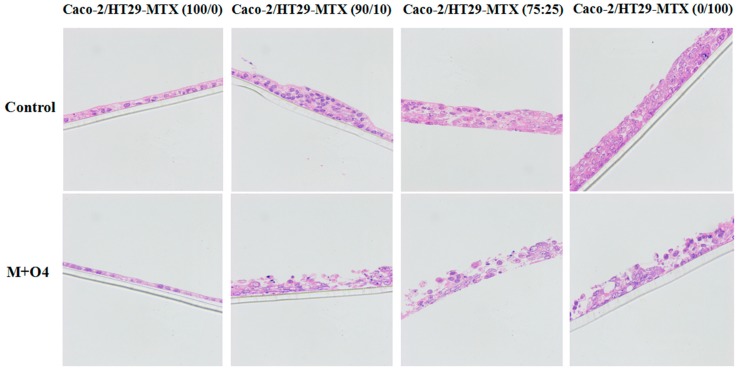
Staining of cell layers, Caco-2 and HT29-MTX co-cultures (100/0, 90/10, 75/25 and 0/100) were stained by hematoxylin-eosin after exposed to the mixture of AFM1 and OTA at 4 μg/mL. Chromatin and cytoplasmic nucleic acids in the nucleus were stained purple blue, components in the cytoplasm and extracellular matrix were stained red. The thickness of the polycarbonate membrane was 20 μm. M+O4 represents AFM1+OTA at 4 μg/mL.

To evaluate the effect of mycotoxins on the cell layer structures, we used hematoxylin–eosin (HE) to stain mono- and co-cultures in the mixtures of AFM1 and OTA at 4 μg/mL. The selection of mycotoxin concentration was based on the above experimental results (Figure 1). The apical side of the Caco-2 single culture showed the thinnest layer as compared with other mono- or co-cultures upon treatment with mycotoxins. The cellular tight connections were severely damaged in the mixtures of AFM1 and OTA treatment at 4 μg/mL (Figure 2). At the same time, the number of cells decreased significantly in all Caco-2/HT29-MTX mono- and co-cultures and it was consistent with the results in Section 2.1.

### 2.3. Effects of AFM1 and OTA Individually Or Collectively on Caco-2/HT29-MTX TEER Values (100/0, 90/10, 75/25, and 0/100)

**Figure 3:**
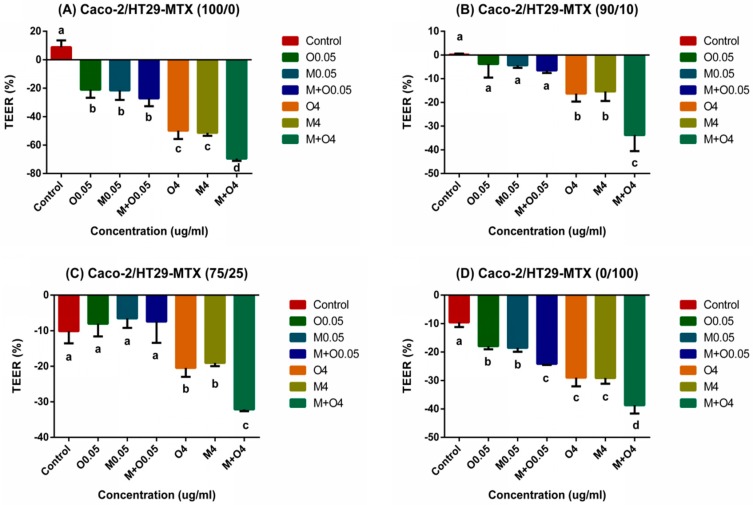
Changes in transepithelial electrical resistance (TEER) values in differentiated Caco-2/HT29-MTX (**A**) 100/0, (**B**) 90/10, (**C**) 75/25, and (**D**) 0/100 co-cultures after treatment with different concentrations of AFM1 and OTA individually or collectively (AFM1+OTA) for 48 h. Results were expressed as the percentage of the difference to the initial value for each insert and are means ±SEM (*n* = 3), respectively. Different letters (a–d) indicate significant differences in TEER values (*p* < 0.05). M0.05 represents AFM1 at 0.05 μg/mL, M4 represents AFM1 at 4 μg/mL, O0.05 represents OTA at 0.05 μg/mL, O4 represents OTA at 4 μg/mL, M+O0.05 represents AFM1+OTA at 0.05 μg/mL, M+O4 represents AFM1+OTA at 4 μg/mL.

Transepithelial Electrical Resistance (TEER) value is one of the important parameter used to study the intestinal barrier integrity. The initial TEER values (before the mycotoxin treatment) of 100/0, 90/10, 75/25, and 0/100 (Caco-2/HT29-MTX) cultures varied from 1078 to 1155 Ω × cm^2^, 320 to 359 Ω × cm^2^, 145 to 178 Ω × cm^2^, and 154 to 176 Ω × cm^2^, respectively. After 48 h of exposure to mycotoxins, the TEER values of the Caco-2/HT29-MTX 100/0 and 0/100 cultures significantly decreased (*p* < 0.01). The 90/10 and 75/25 co-cultures showed non-cytotoxicity at concentrations of 0.05 μg/mL (*p* > 0.05, Figure 3). Similar to the cell viability, the damage caused by mixtures of AFM1 and OTA at 4 μg/mL was the greatest. Individual AFM1 and OTA showed similar cytotoxicities with no significant difference (*p* > 0.05), and the combination cytotoxicity was significantly higher than the cytotoxicity of each alone (*p* < 0.01) at the same concentration.

### 2.4. Effects of AFM1 and OTA Individually Or in Combination on Mucin Gene Expression of Caco-2/HT29-MTX Cultures (100/0, 90/10, 75/25, and 0/100)

**Figure 4:**
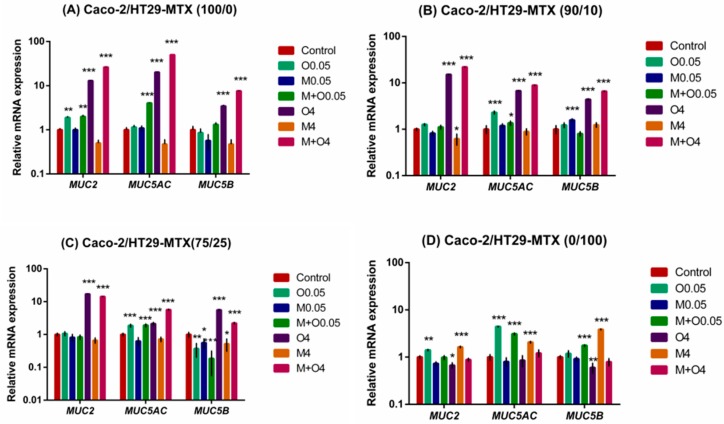
Relative levels of *MUC2*, *MUC5AC* and *MUC5B* mRNA from Caco-2/HT29-MTX (**A**) 100/0, (**B**) 90/10, (**C**) 75/25, and (**D**) 0/100 co-cultures exposed to individual or combinations of AFM1 (0.05, 4 μg/mL), OTA (0.05, 4 μg/mL), or AFM1+OTA (0.05, 4 μg/mL) for 48 h. The expression of mucin genes (*MUC2*, *MUC5AC*, and *MUC5B*) in Caco-2/HT29-MTX co-cultures was quantified by SYBR green quantitative polymerase chain reaction (qPCR) assay. Results are expressed as a percentage of the control and are means ±SEM (*n* = 2). *, **, *** *p* < 0.05, 0.01, and 0.001, respectively, compared with control. M0.05 represents AFM1 at 0.05 μg/mL, M4 represents AFM1 at 4 μg/mL, O0.05 represents OTA at 0.05 μg/mL, O4 represents OTA at 4 μg/mL, M+O0.05 represents AFM1+OTA at 0.05 μg/mL, M+O4 represents AFM1+OTA at 4 μg/mL.

The effects of individual AFM1 and OTA and their mixtures on the expression of *MUC2*, *MUC5AC* and *MUC5B* mRNA were assessed. Results show that the mRNA expression levels of *MUC2*, *MUC5AC* and *MUC5B* were significantly up-regulated upon treatment with OTA alone or a mixture of AFM1 and OTA at 4 μg/mL for most monocultures and co-cultures except HT29-MTX monocultures (Figure 4). In addition, the mRNA expression of *MUC2* and *MUC5AC* in the Caco-2 monoculture was significantly up-regulated upon treatment with OTA alone or its mixture with AFM1 at a lower concentration (0.05 μg/mL, Figure 4A).

For 90/10 co-cultures, there was a significant increase in the expression of *MUC2* mRNA after exposure to 4 μg/mL AFM1 alone as compared with the control. *MUC5AC* mRNA expression levels were significantly up-regulated upon treatment with OTA alone or with OTA combined with AFM1 at 0.05 μg/mL. At 0.05 mg/mL AFM1 alone, *MUC5B* mRNA expression was significantly up-regulated (Figure 4B).

In 75/25 co-cultures, individual and combined AFM1 and OTA significantly affected *MUC5B* mRNA expression. AFM1 alone (0.05 and 4 μg/mL), OTA alone, and the combination of AFM1 and OTA at 0.05 μg/mL caused down-regulation of *MUC5B* mRNA expression, and we observed significant up-regulation of *MUC5AC* mRNA levels in OTA alone and mixtures of AFM1 and OTA at a lower concentration (0.05 μg/mL) as compared with respective controls (Figure 4C).

In the HT29-MTX monoculture, significant up-regulation of *MUC5AC* and *MUC2* mRNA levels was found with OTA alone at 0.05 μg/mL, while significant down-regulation of *MUC5B* and *MUC2* mRNA levels was observed with OTA alone at 4 μg/mL (Figure 4D). AFM1 alone at 4 μg/mL or mixed with OTA at 0.05 μg/mL resulted in a significant up-regulation of *MUC5AC* and *MUC5B* mRNA expression levels (Figure 4D).

### 2.5. Effects of AFM1 and OTA Individually Or in Combination on Mucin Protein Abundance of Caco-2/HT29-MTX Cultures (100/0, 90/10, 75/25, and 0/100)

**Figure 5:**
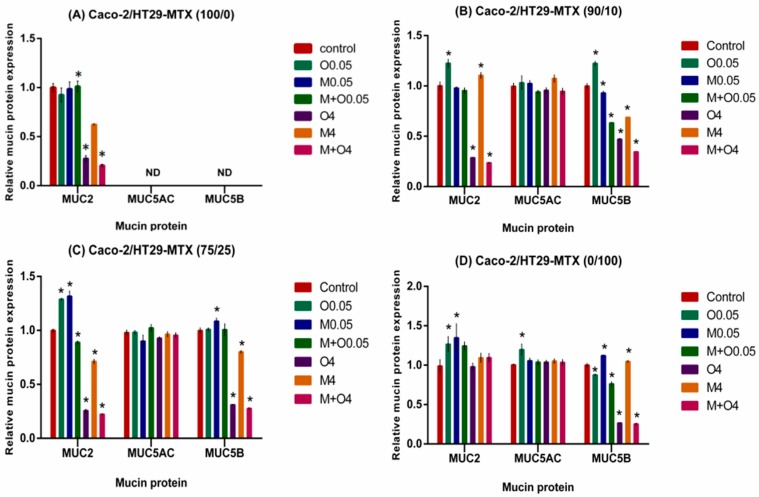
Relative abundance of MUC2, MUC5AC and MUC5B protein in cell supernatants from Caco-2/HT29-MTX (**A**) 100/0, (**B**) 90/10, (**C**) 75/25, and (**D**) 0/100 co-cultures exposed to individual or combinations of AFM1 (0.05, 4 μg/mL), OTA (0.05, 4 μg/mL), or AFM1+OTA (0.05, 4 μg/mL) for 48 h. Relative levels of mucin proteins in the cell supernatant were measured by using human mucin 2, mucin 5 subtype B, and mucin 5 subtype AC ELISA kits. Results are expressed as a percentage of the control and are means ±SEM (*n* = 3). *, **, *** *p* < 0.05, 0.01, and 0.001, respectively, compared with control. M0.05 represents AFM1 at 0.05 μg/mL, M4 represents AFM1 at 4 μg/mL, O0.05 represents OTA at 0.05 μg/mL, O4 represents OTA at 4 μg/mL, M+O0.05 represents AFM1+OTA at 0.05 μg/mL, M+O4 represents AFM1+OTA at 4 μg/mL.

In cell supernatants of most monocultures and co-cultures except HT29-MTX monocultures, OTA alone or combined with AFM1 at 4 μg/mL significantly decreased the MUC2 protein levels, whereas treatment with a combination of AFM1 and OTA at 0.05 μg/mL significantly increased them (Figure 5). There was almost no difference between MUC5AC protein levels in the mycotoxin treatment and controls in all individual cultures or co-cultures. Significant decrease of MUC5B protein levels was noted in the treatment of most monocultures and co-cultures, except Caco-2 monocultures, with OTA alone or combined with AFM1 at 4 μg/mL (Figure 5).

In the 90/10 co-cultures, significant increases in the MUC2 and MUC5B protein levels were observed in cell supernatants after treatment with OTA alone (0.05 μg/mL) or AFM1 alone (4 μg/mL). In addition, AFM1 alone or mixed with OTA resulted in significant increase in MUC5B protein levels at 0.05 μg/mL (Figure 5B).

For the 75/25 co-cultures, the MUC2 protein levels were increased at low concentrations (0.05 μg/mL) and decreased at high concentrations. Similarly, MUC5B protein levels were increased at low concentrations of AFM1 (0.05 μg/mL) and decreased at 4 mg/mL (Figure 5C).

In the HT29-MTX monoculture with mycotoxins alone at 0.05 μg/mL, significant increase of MUC2 protein levels was noted. AFM1 alone and mixtures of AFM1 and OTA at 0.05 μg/mL resulted in a significant increase of MUC5B protein levels, but we found reductions with OTA alone and OTA mixed with AFM1 at 0.05 μg/mL (Figure 5D).

### 2.6. The Interactive Effects of the Combination of AFM1 and OTA on Caco-2/HT29-MTX Single Cultures and Co-Cultures (100/0, 90/10, 75/25, and 0/100)

**Figure 6:**
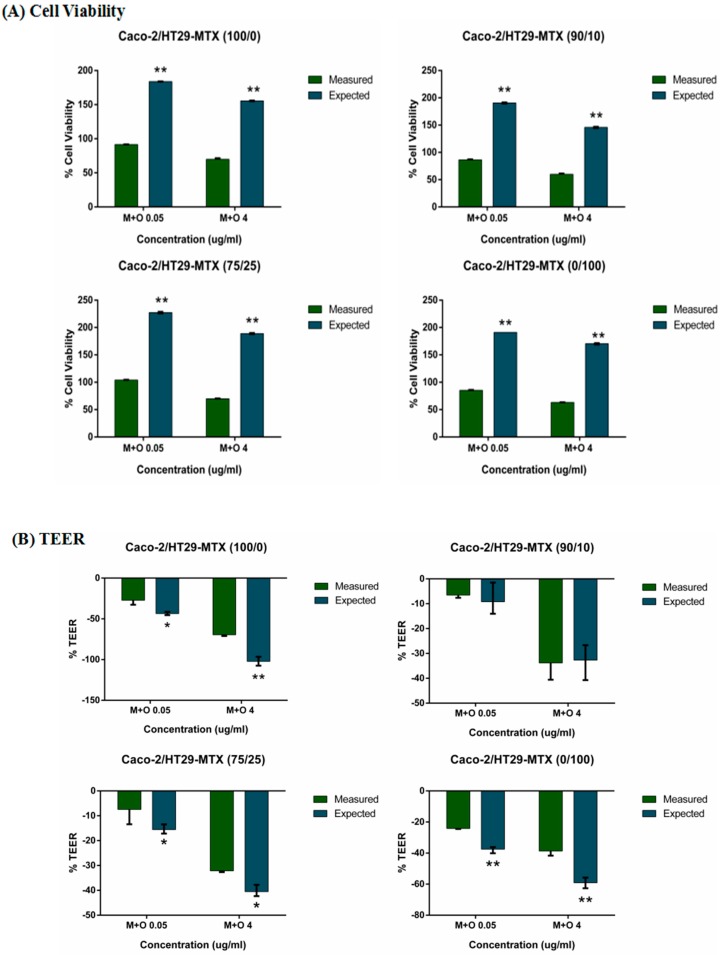

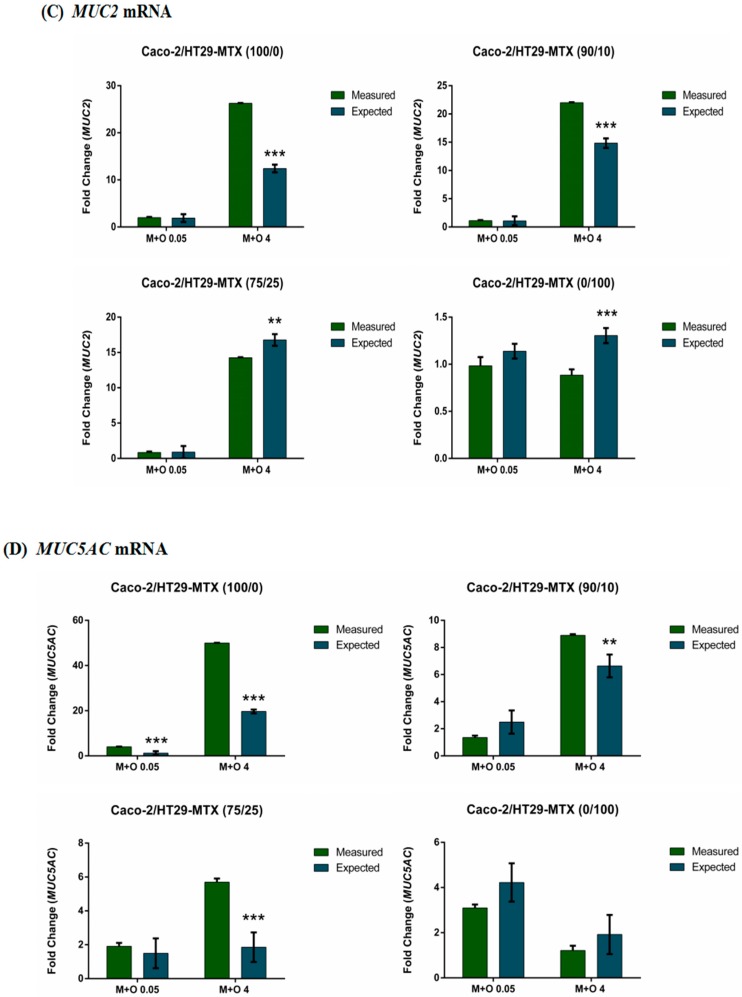

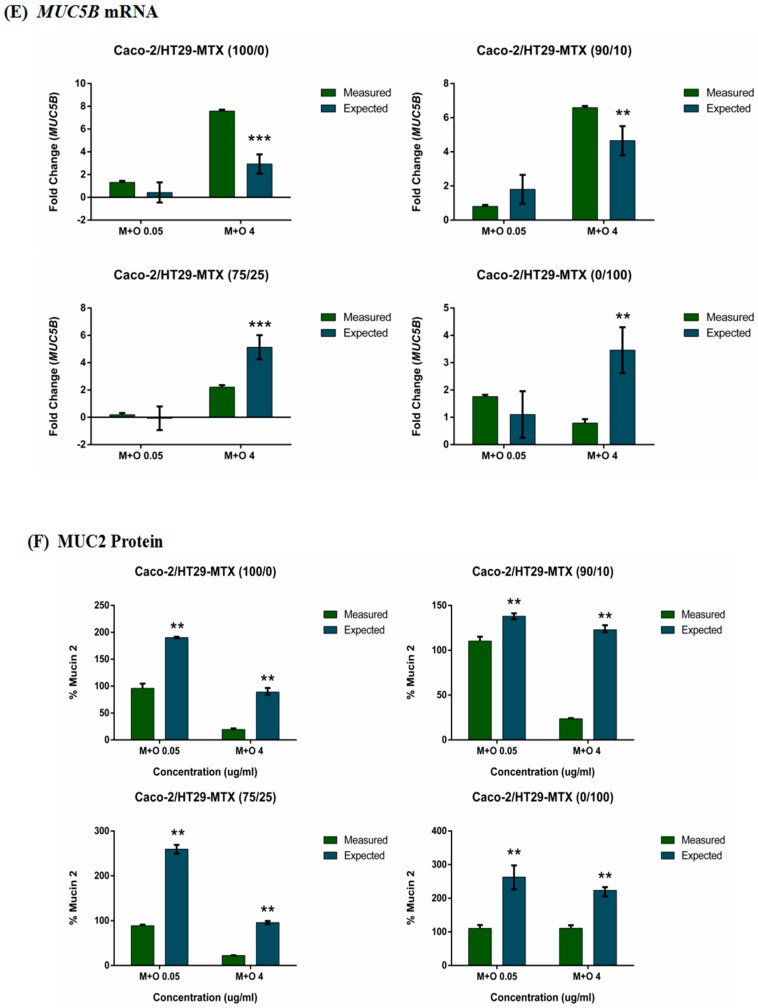

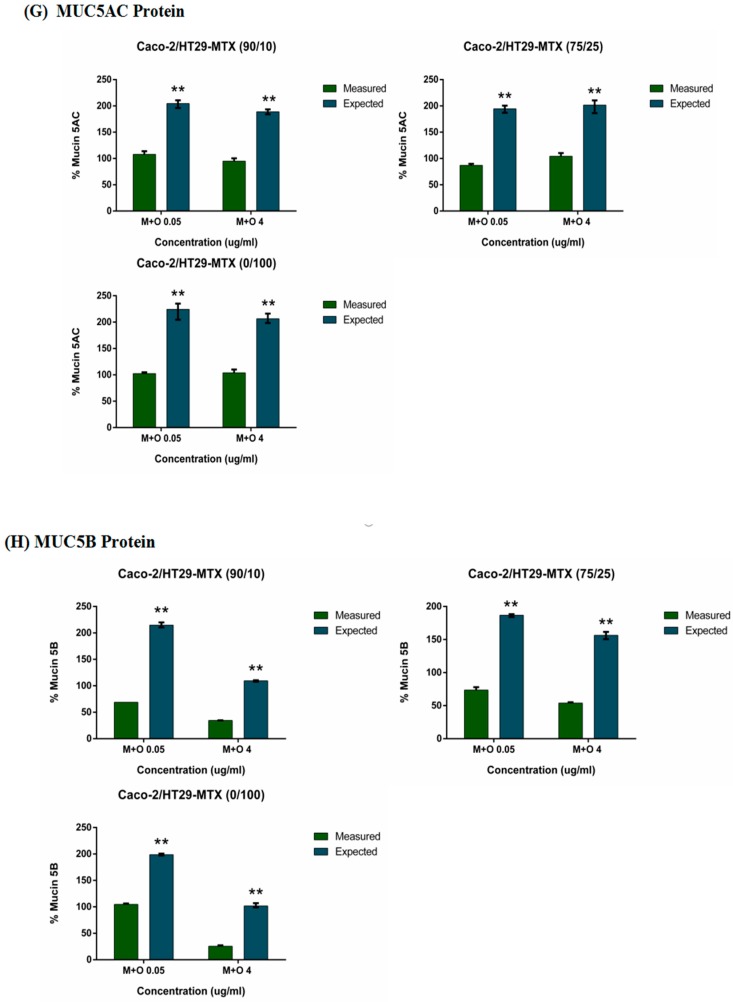
Interactive cytotoxic effects of binary combinations of AFM1 and OTA in (**A**) Cell viability, (**B**) TEER, (**C**) *MUC2* mRNA, (**D**) *MUC5AC* mRNA, (**E**) *MUC5B* mRNA, (**F**) MUC2 protein, (**G**) MUC5AC protein and (**H**) MUC5B protein of Caco-2/HT29-MTX (100/0, 90/10, 75/25, 0/100) co-cultures isolated after 48 h. Data are expressed as a percentage of the untreated control for each parameter. * *p* < 0.05; ** *p* < 0.001; represent both significant synergistic and antagonist effects.

Results for the cell viability show that the mixtures of AFM1 and OTA (0.05 and 4 μg/mL) caused synergistic effects on all Caco-2/HT29-MTX mono- and co-cultures (Figure 6A). An additive effect was found in the TEER of Caco-2/HT29-MTX 90/10 co-cultures, with a non-significant difference (*p* > 0.05) between the measured and expected values. An antagonistic effect was observed in other mono- or co-cultures treated with the mixtures of AFM1 and OTA at 0.05 and 4 μg/mL (Figure 6B). Additive effects were evident at the low concentration (0.05 μg/mL) in all Caco-2/HT29-MTX mono- and co-cultures, antagonistic effects were found in 100/0 and 90/10 cultures at 4 μg/mL, and a synergistic effect on *MUC2* and *MUC5B* mRNA expression was observed in 75/25 and 0/100 cultures at 4 μg/mL (Figure 6C,E). For *MUC5AC* mRNA expression, there was an antagonistic effect in 100/0 cultures and an additive effect in 0/100 cultures at two concentrations of the mixtures. Both 75/25 and 90/10 Caco-2/HT29-MTX cultures showed an additive effect at the low concentration (0.05 μg/mL) and synergistic effects at all protein levels (MUC2, MUC5AC, and MUC5B) after treatment with the mixtures of AFM1 and OTA at 0.05 and 4 μg/mL (Figure 6F–H).

### 2.7. Correlations between Cell Viability, TEER, Mucin mRNA Expression and Mucin Protein Levels

**Figure 7:**
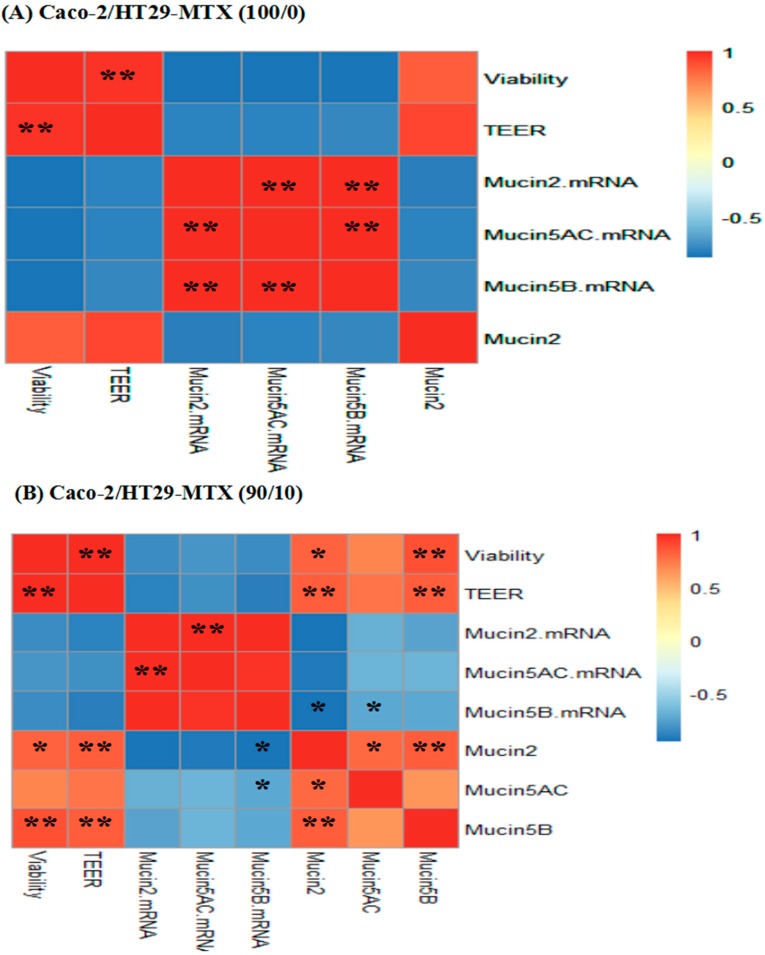

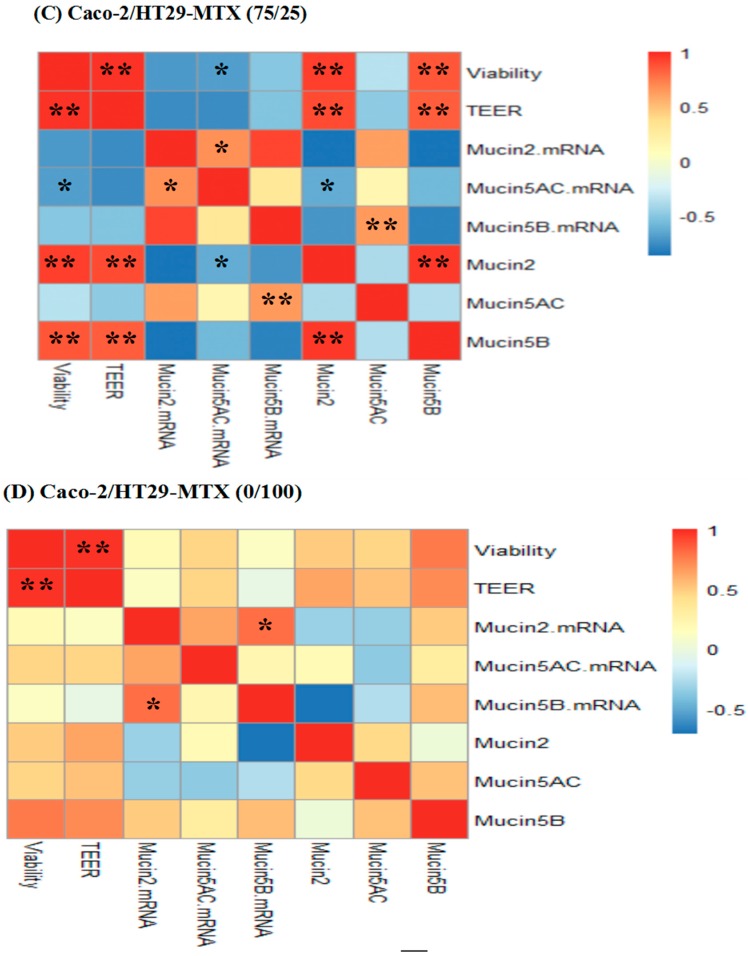
Heat map showing correlations among Cell viability, TEER, Mucin mRNA (*MUC2*, *MUC5AC*, *MUC5B*) and Mucin protein (MUC2, MUC5AC, MUC5B) expression of Caco-2/HT29-MTX (**A**) 100/0, (**B**) 90/10, (**C**) 75/25, and (**D**) 0/100 co-cultures. The heat map is a visual representation of correlated values between each pair of parameters denoted by the corresponding row and the column of the matrix. Red represents a positive correlation, yellow represents a low correlation, and blue represents a negative correlation, as shown in the color key. Statistical significance was analyzed by Spearman’s correlations. The number scale to the right represents the correlation coefficients. The higher the number, the higher the correlation. * *p* < 0.05 and ** *p* < 0.001.

We evaluated the correlations between cell viability values, TEER values, mucin mRNA expression, and mucin protein levels. The results show a significant positive correlation (*p* < 0.01) between cell viability and TEER values in all individual cultures and co-cultures. For the Caco-2 monocultures, there were significant positive correlations between *MUC2*, *MUC5AC*, and *MUC5B* mRNA expression (*p* < 0.01). Significant positive correlations (*p* < 0.01) between *MUC2* and *MUC5AC* mRNA expression were also found for the 90/10 and 75/25 co-cultures. *MUC2* mRNA expression was positively correlated with *MUC5B* mRNA expression (*p* < 0.05) for the HT29-MTX monocultures (Figure 7). In addition, cell viability values, TEER values, as well as MUC2 and MUC5B protein levels, showed significant positive correlations for both 90/10 and 75/25 co-cultures.

## 3. Discussion

Many studies have demonstrated coexisting mycotoxins in nature. Most of these studies, however, focused on the effects of mycotoxins commonly found in cereals and feeds [24,25]. Toxicological data on mycotoxins in milk is limited. This is the first report on AFM1 and OTA existing in milk alone or in combination, which can disrupt the intestinal epithelial barrier and the expression of mucin. We used different initial seeding ratios for Caco-2/HT29-MTX co-cultures that mimic closely the human small (90/10) and large (75/25) intestine tissues [26,27]. Our findings further suggest that there were additive, synergistic, and antagonistic effects between AFM1 and OTA with varying degrees of positive or negative correlation at different test endpoints when mycotoxins damaged the intestinal barrier.

The important parameters, cell viability and TEER values, were used to assess intestinal epithelial cell activity and intestinal barrier integrity. Our results indicate that cell viability and TEER values were significantly reduced in a dose-dependent manner upon exposure to AFM1 and OTA, section staining also showed that mycotoxins caused severely damage to the cells and it may lead directly to a decrease in the number of cells which can produce mucin (Figure 2). In addition, the decrease in cell viability was significantly positively correlated with the decrease in TEER values (Figure 7). However, as reported before [28,29], the increase in cell viability was observed in the 75/25 co-culture at 0.05 µg/mL. It may be attributed by the higher toxin tolerance in large intestine. Moreover, the toxicity of OTA as shown by the cell viability and TEER values was similar to that of AFM1. Taken together, these suggest that the reduced cell viability induced by AFM1 and OTA play a key role in the change in intestinal cell permeability. Other similar studies have also reported this phenomenon. It is widely confirmed that altered intestinal permeability is a major factor contributing to the predisposition to intestinal inflammatory diseases and diarrhea [30]. Therefore, it is reasonable to assume that like AFM1, OTA in milk is a major risk factor.

The mucin secreted by intestinal goblet cells constitutes the main component of the intestinal mucus layer, which forms a mucus layer with water and covers the epithelial free surface, providing lubrication and antagonizing the intestinal adhesion and invasion of pathogenic bacteria [31]. According to the results we obtained, the Caco-2 monolayer did not produce MUC5AC and MUC5B proteins in cell supernatants. This is probably because the Caco-2 monolayer does not have the complete functions of intestinal goblet cells, unlike HT29-MTX cells, which are capable of secreting mucin [32,33]. The protein abundance of MUC5AC was approximately at the lowest limit of detection in the other cell cultures (100/0, 90/10, 75/25), so that there was no significant difference in MUC5AC protein production as compared with that of the respective control group. Two reasons may explain this phenomenon. The first is that the addition of cell-culture supernatants to ensure adequate nutrition of the cells resulted in dilution of the protein. The MUC5AC mucin epitope was not recognized by the monoclonal antibody that we used [34]. Intestinal MUC5AC is usually expressed in small pit cells that secrete mucus in the stomach glands, but expression in colonic tumors cells depends on culture conditions [35]. In addition, our results suggest that mycotoxins eventually lead to down-regulation of mucin expression. In other studies, mycotoxins resulted in a reduced number or proliferation of intestinal goblet cells and up- or down-regulation of intestinal mucin abundance [36,37]. The mucin expression up-regulation may be due to the mechanism by which mycotoxin stimulates the protection of or damage to the intestinal mucosal barrier. It may also be due to the different mycotoxins, dose, time, and experimental conditions. Further research is required to be able to clarify. Mycotoxins mainly affect intestinal mucin through two mechanisms: (i) directly act on intestinal mucin. It affect gene expression level and protein abundance of mucins, and ultimately change the composition and function of mucus layer [37,38]; and (ii) first damage the tight junction of intestinal cells, causing bacteria and other harmful substances to activate cytokines (IL-1, IL-6, IL-8, TNF-a, and IFN-γ) and cellular signaling pathways (MAPK, PKR, JNK, and NF-κB) to affect intestinal mucin [39,40].

There was a significant correlation between mRNA expression levels of different mucins in this study. This may be explained by the fact that MUC2, MUC5AC and MUC5B are all encoded within the same cluster at chromosome position 11p15.5 and that they have some of the same transcriptional characteristics such as the same transcriptional orientation, similarity in size, and distribution of exons [41]. Therefore, similar changes between *MUC2*, *MUC5AC*, and *MUC5B* in our study may occur through multiple mRNA interactions and signaling pathways or other functional relationships [42]. Consistent with previous reports from other researchers [28], we found that mycotoxins made the mRNA change much more than the protein level. There were low correlations between mucin mRNA and the corresponding mucin protein abundances (MUC2, MUC5AC, and MUC5B), as some reports have demonstrated [32,43]. The relationship between protein and mRNA expression levels illustrates the combined results of translation and protein degradation, which are key factors in the regulation of mRNA expression in addition to transcription and mRNA stability [44]. In this study, the differences in mucin mRNA and protein secretion may be due to several reasons: (1) The quantification of mRNA transcription levels is more sensitive than are protein identification and quantification methods [32]. (2) The synthesis and secretion of cellular mucin proteins is not only regulated at the transcriptional level but is also partially or mainly regulated by the cellular abundance of proteins regulated by post-transcriptional or translational regulatory mechanisms [45]. (3) To maintain the relative homeostasis of mRNAs and proteins, such as during exposure to mycotoxins, the production of mucin is reduced. Thus, the cells promote the expression of mucin mRNAs through certain mechanisms to promote the secretion of mucin, or just the opposite situation. (4) Sampling and detection of the position and time point of mucin protein production and mRNA expression are not synchronized, possibly resulting in intracellular mRNA transcription reaching the highest level, while protein levels do not in the cell supernatant. Therefore, *MUC5AC*, and *MUC5B* mRNA expression is observed despite a significant up-regulation of the *MUC2*. The same resultant change in MUC2, MUC5AC, and MUC5B protein levels is not necessarily detected.

Mycotoxins that interact in a synergistic manner are more worrying in terms of risk assessment [46]. In general, synergistic or additive effects occur when mycotoxins with the same model of action and/or the same cellular target coexist [47]. The results also indicate that in all Caco-2/HT29-MTX mono- and co-cultures, the mixtures of AFM1 and OTA (0.05 and 4 μg/mL) caused synergistic effects in the assessment of cell viability and mucin protein levels. This is because AFM1 and OTA may be readily incorporated into cell membranes because of their lipophilic structure. They exert cytotoxicity in a synergistic manner at low or high concentrations [48]. Furthermore, the antagonistic effects of AFM1 and OTA on the TEER and mucin mRNA expression may be related to competition for glutathione in cells [49], and the antagonistic effect shown in the Caco-2/HT29-MTX 90/10 co-culture but the synergistic effect shown in 75/25 may be related to the seeding ratio of the two cells. In the present study, the seeding ratio of Caco-2 cells caused a decrease in the absorption of AFM1 and OTA, other results also indicate that the characteristics of Caco-2/HT29-MTX 90/10 co-culture is similar to Caco-2 mono-culture, but Caco-2/HT29-MTX 75/25 co-culture is similar to HT29-MTX mono-culture [23,26,50]. However, it needs further exploration. The interaction between AFM1 and OTA depends on the time, the concentration and type of mycotoxins, the type of experimental models selected, and the endpoint of the assessment [47]. Nevertheless, we have only simulated and evaluated the coexistence of AFM1 and OTA at the theoretical level, so it is necessary to conduct more investigations to evaluate the real concentration and mechanism of mycotoxin coexistence in milk.

Intestinal mucosal damage caused by contact with high concentrations of mycotoxins and the chances of the body being exposed to exogenous chemicals and pathogens are greatly increased [51]; these may lead to intestinal inflammation, cancer, and other diseases. Intestinal mucin acts as a main component of the intestinal mucosal barrier and plays an important role in the mechanism of mycotoxin-induced intestinal inflammation and cancer [52]. We demonstrated that the combination of AFM1 and OTA significantly altered intestinal cell viability, barrier integrity, as well as mucin expression and mucin production. OTA alone at 4 μg/mL and the mixtures of AFM1 and OTA significantly inhibited the production of mucin MUC2 and MUC5B. These results will help in identifying the potential molecular mechanisms by which mycotoxins AFM1 and OTA affect intestinal mucin expression and production. They also reveal that the toxicity of OTA is at least similar to that of AFM1. They suggested that we should not only pay attention to the coexistence and interaction of mycotoxins in milk, but also need to make a more comprehensive toxicity comparison between OTA and AFM1, which will help establish the OTA limit standard in milk and provide more information that is useful for risk assessment of milk mycotoxins.

## 4. Materials and Methods

### 4.1. Mycotoxin Treatment

AFM1 (structural formula: C17H12O7; molecular weight: 328) and OTA (structural formula: C20H18ClNO6; molecular weight: 403) used in the experiment were purchased from J&K Chemical Ltd. (Shanghai, China). AFM1 and OTA were dissolved in methanol to concentrations of 400 and 5000 μg/mL, respectively, and stored at −20 °C.

Stock solutions of individual mycotoxins were prepared as above. For all cell-based assays, the stock solutions were diluted with serum-free Dulbecco’s modified Eagle medium (DMEM) until the desired concentrations of AFM1 (0.05 μg/mL, 4 μg/mL), OTA (0.05 μg/mL, 4 μg/mL), and their mixed solution (AFM1 + OTA = 0.05 + 0.05 μg/mL, 4 + 4 μg/mL) for the assay were obtained. The control was serum-free medium with methanol at the same concentration as the test article. All of the toxins in the test were processed for 48 h.

### 4.2. Cell Lines and Culture Conditions

The human colorectal adenocarcinoma cell line Caco-2 at passage 18 was obtained from the American Type Culture Collection (ATCC, Manassas, VA, USA); cells at passages 28 to 33 were used in experiments. The HT29-MTX cells were kindly provided by Huiying Li (School of Life Science, Tsinghua University); those used in experiments were from passages 28 to 39. Cells were routinely maintained at 37 °C, in a 95% air/water saturated atmosphere with 5% CO_2_, using complete medium consisting of DMEM with 10% fetal bovine serum (FBS), 1% antibiotics (100 units/mL penicillin and 100 μg/mL streptomycin) and 1% non-essential amino acids (Life Technologies, Carlsbad, CA, USA). Before the required passages of the test were reached, cells were sub-cultured using trypsin-EDTA solution (0.25%), and the complete medium was changed every other day. For Caco-2/HT29-MTX co-cultures, Caco-2 and HT29-MTX cells were grown separately in cell culture dishes (Corning, New York, NY, USA). Cells confluent within 2–4 days were treated with mycotoxins at day 14 using conditions described below for each assay. Each set of experiments used all four co-culture conditions (Caco-2/HT29-MTX: 100/0, 90/10, 75/25, and 0/100) with cells maintained under identical conditions.

### 4.3. Cell Viability Assay

To determine the cytotoxicity of the individual and combined mycotoxins, all proportions of cells (Caco-2/HT29-MTX: 100/0, 90/10, 75/25, and 0/100) were seeded in 1 × 10^5^ cells/well using 100 μl of complete proliferation medium in 96-well plates (Corning). The effects of mycotoxins on the proliferation of each model were then determined by using an Enhanced Cell Counting Kit-8 (CCK-8) (Beyotime Biotechnology, Shanghai, China) according to the manufacturer’s instructions. The absorbance was measured at 450 nm using an automated ELISA reader (Thermo Scientific, Waltham, MA, USA). Results were expressed as the percentage of cell survival rate (%) with respect to the control. Experiments were undertaken in triplicate (three successive passages of cells), each with ten replicates per treatment.

### 4.4. Cell Layer Staining

Caco-2/HT29-MTX cultures (100/0, 90/10, 75/25, and 0/100) were cultured in 24-well transwell chambers (Corning) as described above. After 14 days of culture, all single cultures and co-cultures were washed with Hank’s Balanced Salt Solution (HBSS) two to three times and subsequently fixed with cold methacarn (60% methanol, 30% chloroform, and 10% acetic acid) to preserve the mucus layer [53]. Paraffin-wrapped polycarbonate membranes were cut into 20 μm. Paraffin sections of cells were dehydrated and stained with hematoxylin solution followed by eosin solution. After several washings with water, they will be dehydrated and cleared. Cross sections were mounted on a slide and examined using an inverted Zeiss Axioskop 40 multi-head microscope (Carl Zeiss, Jena, Germany).

### 4.5. TEER Measurement

Caco-2/HT29-MTX co-cultures (100/0, 90/10, 75/25, and 0/100) were cultured in 24-well transwell chambers (Corning) at a density of 1 × 10^5^ cells/well, and the medium was replaced every other day for 14 days. After the TEER values were measured according to the instructions, all cells were challenged for 48 h in non-supplemented media (DMEM only; no FBS or antibiotic) or in medium containing individual and combined AFM1 and OTA. The TEER values after toxin treatment were then measured again, the difference of TEER values before and after 48 h were calculated and the final results were shown as its proportion to the initial values. The determination of the TEER was carried out by a Millicell-ERS volt-ohm meter (Millipore, Temecula, CA, USA). Experiments were repeated three times, with five replicates for each treatment, and the results were expressed relative to the initial TEER values for each insert.

### 4.6. Quantification of Mucin Gene Expression

Caco-2/HT29-MTX cells were seeded at 2 × 10^5^ cells/well in six-well culture plates (Corning). Confluent cells at days 14 were washed with phosphate buffered saline (PBS) buffer and treated with mycotoxins (as described above) in serum-free media for 48 h. The expression of mucin genes (*MUC2*, *MUC5AC*, and *MUC5B*) in Caco-2/HT29-MTX co-cultures was quantified using an SYBR green quantitative polymerase chain reaction (qPCR) kit (Takara, Shiga, Japan); the primer sequences for the quantification of *MUC2*, *MUC5AC*, and *MUC5B* are shown in Table 1.

The total RNA was extracted using RNAiso Plus and was reverse-transcribed into cDNA using a Fast Quantity RT Kit (TIANGEN, Beijing, China) according to the manufacturer’s instructions. Prior to use of the RNA in qPCR, its quality was determined by ensuring values of >1.8 and <2.2 for the A260/A280 ratio. All samples were run on a StepOnePlus real-time PCR system (Applied Biosystems, Foster City, CA, USA). The thermal profile used was 95 °C for 180 s followed by 40 cycles at 95 °C for 3 s and at 60 °C for 30 s.

Relative changes in gene expression levels of *MUC2*, *MUC5AC*, and *MUC5B* induced by mycotoxin treatments were normalized using the 2^−ΔΔ*C*T^ method as described previously. Experiments were repeated two times independently, with each treatment performed in triplicate.

### 4.7. Mucin Protein Abundance

Caco-2/HT29-MTX cells were seeded at 2 × 10^5^ cells/well in six-well culture plates (Corning) and treated with mycotoxins (as described above) in serum-free media for 48 h. Cell-culture supernatants were collected and stored at −80 °C until required for subsequent analyses. Relative levels of mucin proteins in the cell supernatant were measured by using human mucin 2, mucin 5 subtype B, and mucin 5 subtype AC ELISA kits (DLdevelop, Beijing, China) as described in the manufacturer’s instructions. The absorbance was read at 450 nm using an automated ELISA reader (Thermo Scientific, Waltham, MA, USA). The abundance of mucin proteins was calculated in nanograms of mucin protein per milliliter, and the results were expressed as percentages of mucin protein levels (%) with respect to the controls. Experiments were completed in triplicate, each with three replicates per treatment.

### 4.8. Analysis of Interactions and Correlations

Comparison of the measured values with the theoretical expected values, which is based on the measured values, is considered to be reliable. It is also used to evaluate the interaction effect of mycotoxins [54,55]. The expected values were calculated by addition of the mean after exposure to one substance alone (or a mixture of the two substances) to the mean values obtained after exposure to the second or third substance and the calculation of the expected standard error of the mean (SEM) is as follows [47]:

mean (expected for AFM1 + OTA) = mean (AFM1) + mean (OTA) − 100%(1)

SEM (expected for AFM1 + OTA) = ((SEM for AFM1)^2^ + (SEM for OTA)^2^)^1/2^ (2)

The significance of the difference in expected and measured values was calculated using an unpaired t-test, with *p* < 0.05 considered statistically significant.

To analyze the interactive effects of AFM1 and OTA, expected values of cell viability, TEER values, the expression of mucin mRNA, and the production of mucin protein (MUC2, MUC5AC, and MUC5B) were calculated separately. The results were interpreted as follows:

- Additive effects were defined as the measured values for endpoints that were not significantly above or below the expected values (*p* > 0.05).
- Synergistic effects were defined as measured values that were significantly lower than the expected values.
- Antagonistic effects were defined as measured values that were significantly higher than the expected values.
- Correlations among cell viability, TEER values, and the levels of mucin mRNA and protein in Caco-2/HT29-MTX co-cultures treated with AFM1 and OTA individually or collectively were assessed by Spearman’s correlations (nonparametric) and R v3.5.2. (TUNA Team, Tsinghua University, Beijing, China) was used for drawing.

### 4.9. Statistical Analysis

All data analyses were carried out using the SPSS statistical package (SPSS v19.0 for Windows; SPSS Inc., Chicago, IL, USA). Data for cell viability, TEER, mucin mRNA, and protein were expressed as the mean ± standard error of mean of three independent experiments. Differences between groups were analyzed statistically using one-way analysis of variance followed by Tukey’s honestly significant difference test for multiple comparisons. The criterion for significance was established at *p* < 0.05.

## Figures and Tables

**Table 1:** Primer Sequences for Quantification of *MUC2*, *MUC5AC* and *MUC5B* by qPCR.

Primer Set	Product Length (bp)	Forward Primer Sequence (5′-3′)	Reverse Primer Sequence (5′-3′)
*MUC2*	238	AAGACGGCACCTACCTCG	TTGGAGGAATAAACTGGAGAACC
*MUC5AC*	278	GTTTGACGGGAAGCAATACA	CGATGATGAAGAAGGTTGAGG
*MUC5B*	171	GTGACAACCGTGTCGTCCTG	TGCCGTCAAAGGTGGAATAG
GADPH	235	GGAGTCCACTGGCGTCTT	GAGTCCTTCCACGATACCAAA
